# Studies on clinical signs and biochemical alteration in pregnancy toxemic goats

**DOI:** 10.14202/vetworld.2016.869-874

**Published:** 2016-08-18

**Authors:** Prasannkumar R. Vasava, R. G. Jani, H. V. Goswami, S. D. Rathwa, F. B. Tandel

**Affiliations:** 1 Department of Veterinary Medicine, College of Veterinary Science and Animal Husbandry, Anand Agricultural University, Anand - 388 001, Gujarat, India; 2 Department of Veterinary Anatomy, College of Veterinary Science and Animal Husbandry, Anand Agricultural University, Anand - 388 001, Gujarat, India; 3 Department of Veterinary Physiology & Biochemistry, College of Veterinary Science and Animal Husbandry, Anand Agricultural University, Anand - 388 001, Gujarat, India

**Keywords:** alteration, biochemistry, clinical signs, goat, pregnancy toxemia

## Abstract

**Aim:**

This study was planned to reveal the clinical signs and biochemical alterations in pregnancy toxemic goats.

**Materials and Methods:**

Blood samples were collected from 20 healthy pregnant and 45 pregnancy toxemic goats and analyzed biochemically.

**Results:**

The most significant clinical findings were observed in naturally affected goats with pregnancy toxemia included anorexia, recumbency, lethargy, opisthotonos, dropped head, periodic convulsion, sweetish fruity odor from breath, apparent blindness, bloat, grinding of teeth, and frothy salivation. In this study, the level of serum glutamic-oxaloacetic transaminase (SGOT) (84.23±1.44 IU/L), serum glutamic pyruvic transaminase (SGPT) (216.01±4.07 IU/L), blood urea nitrogen (BUN) (22.24±0.31 mg/dl), creatinine (2.13±0.09 mg/dl), β-hydroxybutyric acid (BHBA) (0.46±0.83 mmol/L), and non-esterified fatty acid (NEFA) (1.67±0.71 mmol/L) was significantly higher whereas glucose (30.89±0.38 mg/dl) and calcium (8.10±0.20 mg/dl) levels were significantly decreased in pregnancy toxemic goats as compared to healthy goats.

**Conclusion:**

The goats with pregnancy toxemia exhibited clinical signs include anorexia, recumbency, sweetish fruity odor from breath, apparent blindness, bloat, grinding of teeth, and frothy salivation. Biochemically, there were significantly decreased the level of glucose and calcium, and increased level of SGPT, SGOT, BUN, creatinine, BHBA, and NEFA in the pregnancy toxemic goats.

## Introduction

Pregnancy toxemia, also known as “twin-lamb” disease, is a metabolic disorder of pregnant small ruminants, caused by an abnormal metabolism of carbohydrates and fats, which occurs at the final stage of pregnancy [1]. Obese ewes or does carrying multiple fetuses are at higher risk to develop the disease because of the limited space for adequate intake of feed [2]. Rapid fetal development at the late gestation causes rapid mobilization of the fat stores to assure adequate energy. The liver also increases gluconeogenesis to facilitate glucose availability to the fetus. However, in the negative energy balance (NEB), this increased mobilization may overwhelm the capacity of the liver resulting in hepatic lipidosis. At the same time, ketone bodies are being produced and accumulated, which eventually leads to excessive ketone bodies in blood circulation, and thus increasing the susceptibility to pregnancy toxemia [3].

The early detection of pregnancy toxemia in susceptible animals is essential for successful treatment. In clinical pregnancy toxemia, the diagnosis is based on history, clinical signs of hepatic encephalopathy, and the results of serum biochemical analyses [4].

While clinical pregnancy toxemia in sheep was relatively well studied, there is a paucity of information regarding metabolic changes in the clinical form of the disease, especially in goats. So, this study was planned.

## Materials and Methods

### Ethical approval

Samples were collected from clinical cases coming to Veterinary Hospital at Veterinary College, Anand Agricultural University, Anand. Hence, this particular study did not require ethical approval.

### Study area

Anand district is situated at latitude 22° N and longitude 72° E with 2939.9 km^2^ areas in Gujarat, India, and witnessed a temperature range of 35-38°C with a maximum of 42°C, relative humidity of 57-55% and rainfall about 1-4 inch. The season in this area can be broadly classified into hot and dry summer from March to June, rainy (monsoon) season from July to October, and the winter (mild) season from November to February.

### Study period

The data, recorded from the case records of Department of Teaching Veterinary Clinical Complex, Veterinary College, Anand, were compiled and analyzed for a period of 6-month from June 1, 2015, to December 31, 2015.

### Sample collection

All the cases of pregnancy toxemia were diagnosed using Rothera’s qualitative test from urine samples [5]. The blood samples were collected from jugular vein in 20 healthy pregnant as control and 45 pregnancy toxemic goats to carry out different laboratory and biochemical tests by standard kits. Serum glutamic pyruvic transaminase (SGPT) and serum glutamic-oxaloacetic transaminase (SGOT) were estimated by modified IFCC method (Coral Pvt. Ltd). Glucose, blood urea nitrogen (BUN), calcium, magnesium, phosphorus, and creatinine were estimated by glucose oxidase-peroxidase, diacetylmonoxime, o-cresolphthalein complexone, calmagite, Molybdate U.V., and Mod. Zaffe’s Kinetic method. β-hydroxyl butyric acid (BHBA) was estimated by D-3-hydroxybutyrate kit and non-esterified fatty acid (NEFA) were estimated by NEFA Kit (Randox Laboratories Ltd).

### Statistical analysis

Data, obtained from biochemical parameters, were statistically analyzed by Student’s t-test as per the method described by Snedecor and Cochran [6]. T-test: Two samples assuming unequal variance were used for comparing of pregnancy toxemic and healthy goats biochemical parameters. Variables with p<0.05 were considered as statistically “significant,” variables with p<0.01 were considered as statistically “highly significant,” and variables with p>0.05 were considered as statistically “non-significant.”

## Results

**Figure-1:**
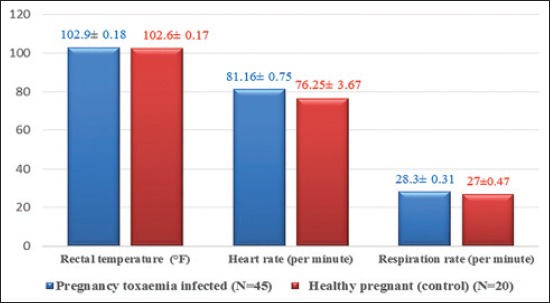
Mean±standard error values of vital signs of healthy pregnant and pregnancy toxemic goats.

**Figure-2:**
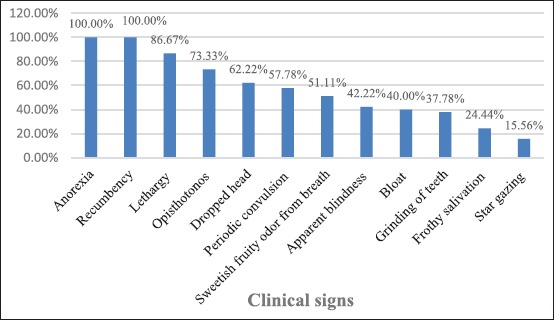
Clinical findings in pregnancy toxemic goats.

The vital signs like rectal temperature and respiration rate were normal, but heart rate was significantly increased than normal goats. The most significant clinical findings were observed in naturally affected goat with pregnancy toxemia included anorexia (100.00%), recumbency (100.00%), lethargy (86.67%), opisthotonos (73.33%), dropped head (62.22%), periodic convulsion (57.78%), sweetish fruity odor from breath (51.11%), apparent blindness (42.22%), bloat (40.00%), grinding of teeth (37.78%), and frothy salivation (24.44%) (Tables-1 and 2; Figures-1 and 2).

**Table-1:** Vital signs observed in pregnancy toxemic and healthy goats.

Parameter (Units)	Pregnancy toxemia infected (N=45)	Healthy pregnant (N=20)
Rectal temperature (°F)	102.9±0.18	102.6±0.17
Heart rate (per minute)	81.16±0.75*	76.25±3.67
Respiration rate (per minute)	28.3±0.31	27±0.47

* p<0.05 (significant), N=Number of goats

**Table-2:** Clinical variant recorded from pregnancy toxemic goats.

Clinical signs	Total number of affected goats shown clinical symptoms (N=45)	Percentage
Anorexia	45	100.00
Recumbency	45	100.00
Lethargy	39	86.67
Opisthotonos	33	73.33
Dropped head	28	62.22
Periodic convulsion	26	57.78
Sweetish fruity odor from breath	23	51.11
Apparent blindness	19	42.22
Bloat	18	40.00
Grinding of teeth	17	37.78
Frothy salivation	11	24.44
Star gazing	07	15.56

N=Number of goats

**Figure-3:**
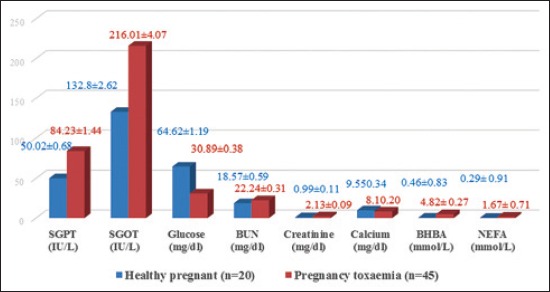
Mean±standard error values of biochemical parameters of healthy pregnant and pregnancy toxemic goats.

The study was aimed to evaluate the biochemical indicators in goats positive for naturally occurring pregnancy toxemia in comparison with normal healthy goats (Table-1). The mean values of SGPT (IU/L), SGOT (IU/L), glucose (mg/dl), BUN (mg/dl), creatinine (mg/dl), calcium (mg/dl), magnesium (mg/dl), phosphorus (mg/dl), BHBA (mmol/L) and NEFA (mmol/L) in 20 healthy pregnant goats were 50.02±0.68 (IU/L), 132.80±2.62 (IU/L), 64.62±1.19 (mg/dl), 18.57±0.59 (mg/dl), 0.99±0.11 (mg/dl), 9.55±0.34 (mg/dl), 3.04±0.14 (mg/dl), 6.91±0.52 (mg/dl), 0.46±0.83 (mmol/L) and 0.29±0.91 (mmol/L), respectively. Among various biochemical parameters evaluated from 45 pregnancy toxemic goats, the mean values of SGPT (84.23±1.44 IU/L), SGOT (216.01±4.07 IU/L), BUN (22.24±0.31 mg/dl), creatinine (2.13±0.09 mg/dl), BHBA 4.82±0.27 (mmol/L), and NEFA 1.67±0.71 (mmol/L) increased significantly in goats with pregnancy toxemia. Whereas, the level of glucose (30.89±0.38 mg/dl) and calcium (8.10±0.20 mg/dl) decreased significantly in goats with pregnancy toxemia (Table-3 and Figure-3).

**Table-3:** Biochemical parameters of healthy pregnant and pregnancy toxemia affected goats.

Parameters	Mean±SE	

	Healthy pregnant (N=20)	Pregnancy toxemia (N=45)
SGPT (IU/L)	50.02±0.68	84.23±1.44**
SGOT (IU/L)	132.80±2.62	216.01±4.07**
Glucose (mg/dl)	64.62±1.19	30.89±0.38**
BUN (mg/dl)	18.57±0.59	22.24±0.31**
Creatinine (mg/dl)	0.99±0.11	2.13±0.09**
Calcium (mg/dl)	9.55±0.34	8.10±0.20**
Magnesium (mg/dl)	3.04±0.14	2.74±0.09
Phosphorus (mg/dl)	6.91±0.52	6.72±0.15
BHBA (mmol/L)	0.46±0.83	4.82±0.27**
NEFA (mmol/L)	0.29±0.91	1.67±0.71**

** p<0.01 (highly significant). N=Number of goats, SGOT=Serum glutamic-oxaloacetic transaminase, SGPT=Serum glutamic pyruvic transaminase, BUN=Blood urea nitrogen, BHBA=β-hydroxybutyric acid

## Discussion

In this study, rectal temperature and respiratory rate were found within normal range, which were contrast to Rodolfo *et al*. [7], who reported increased body temperature. In this study, increased heart rate (81.16±0.75/min) substantiated the findings of Manokaran *et al*. [8], Balikci *et al*. [9], and Reddy *et al*. [10]. Anorexia was observed in all cases which were in agreement with Scott and Woodman [11], El-Sebaie [12], Andrews [13], Balikci *et al*. [9], Barakat *et al*. [14], Al-Qudah [15], Manokaran *et al*. [8], Abdelaal *et al*. [16], Souto *et al*. [17], Reddy *et al*. [10], Rodolfo *et al*. [7], and Rani *et al*. [18]. Depression of feed intake before kidding has been considered a major factor in the development of the pregnancy toxemia. Accumulation of fat in the liver occurred during periods of elevated fat mobilization, especially in goats with excessive fat body reserves at kidding. During this period, incomplete breakdown of NEFA was responsible for the production of ketone bodies. Due to lack of energy and hypocalcemia, there was recumbency in the pregnancy toxemic goat which was in agreement with Scott and Woodman [11], El-Sebaie [12], Andrews [13], Balikci *et al*. [9], Barakat *et al*. [14], Hefnawy *et al*. [19], Al-Qudah [15], Abdelaal *et al*. [16], and Reddy *et al*. [10]. 23 (51.11%) goats presented with sweetish fruity odor from breath. Similar clinical findings in goat with pregnancy toxemia were recorded by Balikci *et al*. [9], Hefnawy *et al*. [20], Abdelaal *et al*. [16], and Rani *et al*. [18]. The sweetish odor which was a typical characteristic of ketone bodies which was increased in pregnancy toxemia. 11 (24.44%) goats presented with frothy salivation were in agreement with Balikci *et al*. [9] and Barakat *et al*. [14]. Chew movement and salivation were seen in the nervous form of pregnancy toxemia in does. The teeth grinding behavior in 17 (37.78%) goats suffered from pregnancy toxemia was in agreement with Balikci *et al*. [9], Abdelaal *at al*. [16], Reddy *et al*. [10], and Rani *et al*. [18]. It was due to the cerebral hypoglycemia which results teeth gnashing behavior. 39 (86.67%) goats presented with lethargy in agreement with Andrews [13] and Reddy *et al*. [10]. It was due to lack of adequate glucose in body for the production of energy. 19 (42.22%) goats presented with apparent blindness were in agreement with El-Sebaie [12], Andrews [13], Barakat *et al*. [14], and Al-Qudah [15]. Nervous degeneration was due to cerebral hypoglycemia resulted into apparent blindness. 22 (57.58%) goats present with convulsion due to nervous manifestation because of hypoglycemia and ketonemia were in agreement with Hefnawy *et al*. [20]. 18 (40.00%) goats presented with bloat were in agreement with Souto *et al*. [17], Rodolfo *et al*. [7], and Rani *et al*. [18] and the bloat was due to longer time recumbency, anorexia, stasis of rumen motility, and ketoacidosis. 28 (62.22%) goats presented with dropped head were in agreement with Abdelaal *et al*. [16] and Reddy *et al*. [10]. Dropped head condition was nervous manifestation due to lack of energy in the nervous form of pregnancy toxemia. 34 (75.56%) goats, presented with the extension of head, were in agreement with Abdelaal *et al*. [16]. 7 (15.56%) goats, presented with stargazing posture due to the nervous degeneration, were in agreement with Andrews [13], Barakat *et al*. [14], and Rani *et al*. [18]. 33 (73.33%) goats presented with opisthotonos were in agreement with Reddy *et al*. [10] and the opisthotonos was due to hypoglycemia, ketonemia, and neurodegeneration.

Increased SGPT and SGOT level of the present study were in agreement with report of Barakat *et al*. [14], Gupta *et al*. [21], Balikci *et al*. [9], Hefnawy *et al*. [19], Abdelaal *et al*. [16], Albay *et al*. [22], Anoushepour *et al*. [23], Reddy *et al*. [10], Marutsova [24], and Abba *et al*. [25]. In contrast with the present study, Gupta *et al*. [21] reported decreased values of SGPT and SGOT. In this study, SGPT and SGOT were increased due to fat mobilization because of NEB and hepatic damage or hepatic lipidosis. Due to energy deficiency, the body used its fatty tissue which reserves as a source of energy and increased response of free circulating free fatty acids that reach to the liver so, subsequent induce fatty infiltration.

Increased BUN and creatinine level were in agreement with report of Kolb and Kaskom [26], Barakat *et al*. [14], Hefnawy *et al*. [19], Lima *et al*. [4], Abdelaal *et al*. [16], Souto *et al*. [17], Anoushepour *et al*. [23], Rodolfo *et al*. [7], and Reddy *et al*. [10]. In this study, BUN and creatinine were increased suggested that involvement of kidney due to catabolism. Increased BUN and creatinine attributed to severe kidney dysfunction accompanied with acidosis which is the result of increased ketone body in general circulation. There was fatty infiltration in tubular epithelium of kidney in pregnancy toxemic goat leads to elevation of both parameters.

Decreased glucose level of the present study was in agreement with result of many researcher such as McClymont and Setchell [27], Robert [28], Lindsay and Pethick [29], Buswell *et al*. [30], Cantley *et al*. [31], El-Sebaie [12], Scott and Woodman [11], Andrews [13], Henze *et al*. [32], Marteniuk and Herdt [33], Gupta *et al*. [21], Barakat *et al*. [14], Ismail *et al*. [34], Schlumbohm and Harmeyer [35], Balikci *et al*. [9], Hefnawy *et al*. [20], Al-Qudah [15], Hefnawy *et al*. [19], Manokaran *et al*. [8], Abdelaal *et al*. [16], Anoushepour *et al*. [23], Albay *et al*. [22], Sharma *et al*. [36], Reddy *et al*. [10], Gurdogan *et al*. [37] and Rani *et al*. [18]. However, in contrast to the present study, report of Souto *et al*. [17] and Lima *et al*. [4] showed hyperglycemia in the later stages of pregnancy toxemia when the fetuses were dead. Hypoglycemia was due to dietary deficiency of net energy along with the increased demand for energy in the later part of pregnancy due to twin or triplet. Due to increased BHBA, there was NEB, which causes hypoglycemic effect, reduced food intake and glucose turnover leads to pregnancy toxemia.

Decreased calcium level of the present findings was in agreement with results of Jopp and Quinlivan [38], Anoushepour *et al*. [23], Hefnawy *et al*. [19], Manokaran *et al*. [8], Souto *et al*. [17], Albay *et al*. [22], Rodolfo *et al*. [7], Reddy *et al*. [10], and Rani *et al*. [18]. Decreased calcium level might be due to high need of calcium for fetal skeleton development. When doe carry twin or triplet in last trimester, there was a greater risk of the development of pregnancy toxemia. Magnesium and phosphorus level were found in the normal range which substantiated the findings of Hefnawy *et al*. [19] and Ismail *et al*. [34]. In contrast to the present study, Souto *et al*. [17] and Rodolfo *et al*. [7] reported decreased phosphorus level in pregnancy toxemia.

Increased BHBA level was in agreement with report of Scott and Woodman [11], Ismail *et al*. [34], Balikci *et al*. [9], Al-Qudah [15], Hefnawy *et al*. [19], Gonzalez *et al*. [39], Abdelaal *et al*. [16], Olfati *et al*. [40], Anoushepour *et al*. [23], Gurdogan *et al*. [37], Sharma *et al*. [14], and Marutsova [24]. Increased NEFA level was in agreement with the report of Marteniuk and Herdt [33], Olfati *et al*. [40], Anoushepour *et al*. [23], and Sharma *et al*. [14]. The increases in the serum BHBA and NEFA level could be attributed to the lipolysis of tissue and the release of long-chain fatty acids, which were converted by the liver into ketones in goats. Moreover, these increased could be attributed to disturbance in carbohydrate and fat metabolism leading to hypoglycemia and mobilization of fat stores which lead to hepatic ketogenesis according to Rook [41] and Hefnawy *et al*. [19].

## Conclusion

The goats with naturally occurring pregnancy toxemia exhibited clinical signs includes anorexia, recumbency, sweetish fruity odor from breath, apparent blindness, bloat, grinding of teeth, and frothy salivation. Characteristic biochemical pattern associated with pregnancy toxemia in goats showed that there were decreased the level of glucose and calcium, and increased levels of SGPT, SGOT, BUN, creatinine, BHBA and NEFA.

In the present surveillance, from June to December, the most of animals which shown the clinical sign of recumbency, were suffered from pregnancy toxemia which was confirmed by biochemical tests and urine analysis.

## Authors’ Contributions

This study was a part of PRV’s original research work during M.V.Sc. thesis program. RGJ had designed the plan of work. HVG and FBT helped during sampling, statistical analysis, and manuscript preparation. SDR helped in the laboratory work. All the authors read and approved the final manuscript.
